# Effectiveness of oseltamivir treatment on clinical failure in hospitalized patients with lower respiratory tract infection

**DOI:** 10.1186/s12879-021-06812-2

**Published:** 2021-10-27

**Authors:** Timothy L. Wiemken, Stephen P. Furmanek, Ruth M. Carrico, Paula Peyrani, Daniel Hoft, Alicia M. Fry, Julio A. Ramirez

**Affiliations:** 1 Division of Infectious Diseases, Allergy, and Immunology, Department of Internal Medicine, Saint Louis University School of Medicine, 1100 South Grand Blvd #817, Doisy Research Center, St. Louis, MO 63104 USA; 2 Division of Infectious Diseases, Department of Medicine, University of Louisville School of Medicine, Louisville, KY USA; 3 Centers for Disease Control and Prevention, Atlanta, Georgia; 4 Present Address: Pfizer Inc., Collegeville, PA USA

**Keywords:** Tamiflu, Flu, Heterogenous treatment effects, Causal forest

## Abstract

**Background:**

Influenza is associated with excess morbidity and mortality of individuals each year. Few therapies exist for treatment of influenza infection, and each require initiation as early as possible in the course of infection, making efficacy difficult to estimate in the hospitalized patient with lower respiratory tract infection. Using causal machine learning methods, we re-analyze data from a randomized trial of oseltamivir versus standard of care aimed at reducing clinical failure in hospitalized patients with lower respiratory tract infection during the influenza season.

**Methods:**

This was a secondary analysis of the Rapid Empiric Treatment with Oseltamivir Study (RETOS). Conditional average treatment effects (CATE) and 95% confidence intervals were computed from causal forest including 85 clinical and demographic variables. RETOS was a multicenter, randomized, unblinded, trial of adult patients hospitalized with lower respiratory tract infections in Kentucky from 2009 through 2012. Adult hospitalized patients with lower respiratory tract infection were randomized to standard of care or standard of care plus oseltamivir as early as possible after hospital admission but within 24 h of enrollment. After randomization, oseltamivir was initiated in the treatment arm per package insert. The primary outcome was clinical failure, a composite measure including failure to reach clinical improvement within 7 days, transfer to intensive care 24 h after admission, or rehospitalization or death within 30 days.

**Results:**

A total of 691 hospitalized patients with lower respiratory tract infections were included in the study. The only subgroup of patients with a statistically significant CATE was those with laboratory-confirmed influenza infection with a 26% lower risk of clinical failure when treated with oseltamivir (95% CI 3.2–48.0%).

**Conclusions:**

This study suggests that addition of oseltamivir to standard of care may decrease clinical failure in hospitalized patients with influenza-associated lower respiratory tract infection versus standard of care alone. These results are supportive of current recommendations to initiate antiviral treatment in hospitalized patients with confirmed or suspected influenza as soon as possible after admission.

*Trial registration* Original trial: Clinical Trials.Gov; Rapid Empiric Treatment With Oseltamivir Study (RETOS) (RETOS); ClinicalTrials.gov Identifier: NCT01248715 https://clinicaltrials.gov/ct2/show/NCT01248715

## Introduction

Influenza is associated with excess morbidity and mortality of individuals each year in the United States and contributes substantially to the national healthcare burden each winter [1]. Neuraminidase inhibitors such as oseltamivir, peramivir, and zanamivir are one of three categories of FDA-approved therapies for influenza illness and reduce duration of infection through prevention of virus exit from infected cells [2]. Because of this mechanism, administration of drug early in the course of infection is most efficacious [3]. We previously conducted a randomized trial to evaluate the impact of oseltamivir on clinical outcomes of hospitalized patients with lower respiratory tract infection associated with influenza [4]. In that study, we found limited, and not statistically significant, efficacy of oseltamivir in reducing clinical failure, a composite measure including failure to reach clinical improvement within 7 days, transfer to intensive care 24 h after admission, or rehospitalization or death within 30 days, in hospitalized patients with influenza-associated lower respiratory tract infection [4]. Although the average treatment effect was not significant, it is possible that therapy had clinical benefits in subgroups of patients, or our analytical approach was insufficient for the data obtained.

Since the results of this study were published, there have been many innovations in analytical approaches for these types of data, specifically the field of machine learning [5]. These advancements have improved not only our ability to develop predictive models but also allow for computation of treatment effects. In this area, treatment effect computation is also possible across subgroups of individuals, with fewer limitations of sample size and false discovery rates that plague frequentist statistical approaches [6]. Since the sample size of influenza virus infected patients in our initial randomized trial was relatively small and we were underpowered for our primary endpoint, it is possible that we were unable to appropriately detect subgroups in which oseltamivir therapy was efficacious.

The objective of this post hoc study was to utilize a novel machine learning method, the causal forest [7], to evaluate subgroups of hospitalized patients with lower respiratory tract infection who may have differential therapeutic response to oseltamivir therapy for prevention of clinical failure.

## Methods

### Design and patients

This was a secondary analysis of the Rapid Empiric Treatment with Oseltamivir Study (RETOS) [4]. Briefly, RETOS was a randomized, unblinded, trial of adult patients hospitalized with lower respiratory tract infections in Kentucky from 2009 through 2012. Patients were randomized to group A (standard of care) or group B (standard of care plus oseltamivir) as early as possible after hospital admission but within 24 h of enrollment. Both per-protocol and intent-to-treat analyses were performed in the original study since all patients with lower respiratory tract infections were randomized regardless of etiology, though subsets with documented influenza virus infection by reverse transcriptase polymerase chain reaction (rt-PCR) were also analyzed. For the purposes of the present study, all patients in the intent-to-treat analysis (randomized patients with lower respiratory tract infection regardless of documented etiology) were included.

### Study variables

**Fig. 1:**
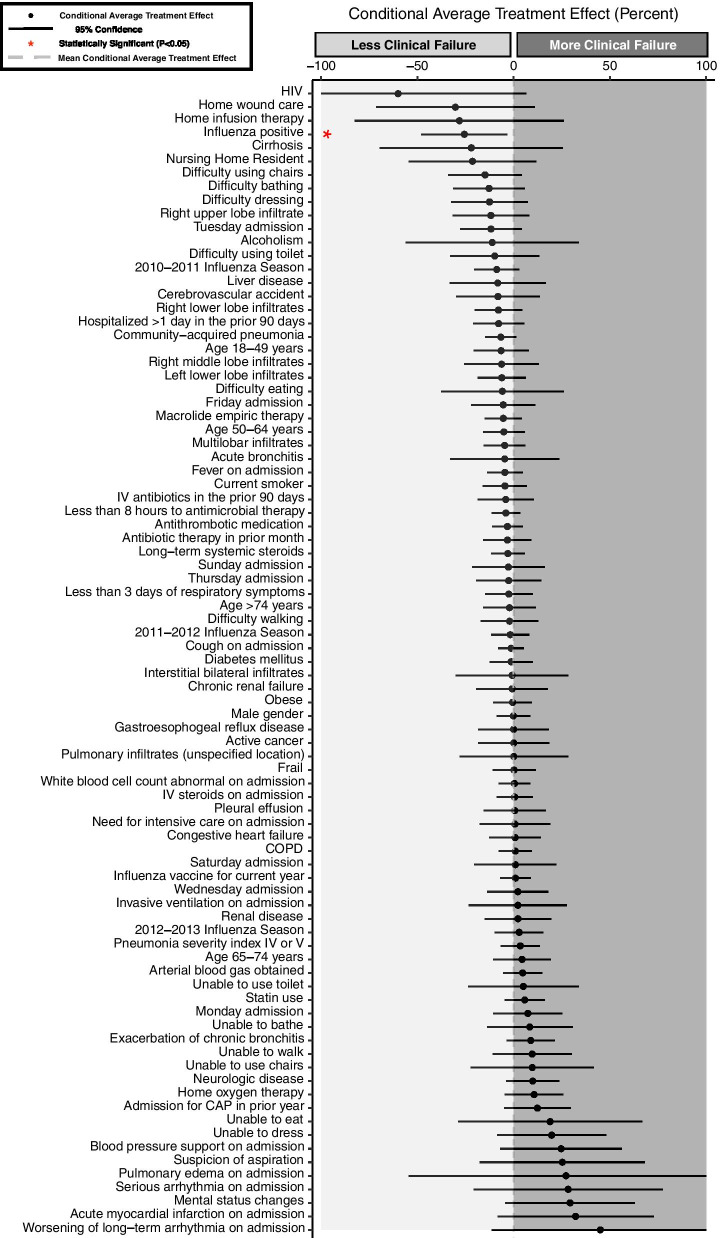
Conditional average treatment effects for clinical failure. Variable listed is the subgroup of patients for which the treatment effect was computed for patients treated versus not treated with oseltamivir

A total of 85 variables were used to investigate potential heterogeneity in average treatment effects between oseltamivir and clinical failure. All variables used in the models are included in Fig. 1 of the results. Variables were selected based on clinical interest, complete data availability in the study database, and potential need for adjustment due to confounding effects in the assessment of treatment effects of oseltamivir on clinical failure.

### Study outcomes

The primary outcome was clinical failure, as defined in our original study: a composite measure including failure to reach clinical improvement within 7 days, transfer to intensive care 24 h after admission, or rehospitalization or death within 30 days.

### Human subjects protection

The University of Louisville Human Subjects Research Protection Program Office (Protocol 10.0465), the Robley Rex VA Medical Center Institutional Review Board (IRB; Protocol 0068/00325), and each participating hospital reviewed and approved the study prior to any enrollment. The Centers for Disease Control and Prevention (CDC) IRB granted reliance on local ethical review approvals as the funding agency of the original study.

### Statistical analysis

Baseline characteristics of patients were compared using Chi-Squared or Fishers Exact tests. Q values were also computed for all variables versus treatment status to account for the increased false discovery rate due to multiple comparison in this analysis. Causal forests were used to estimate conditional average treatment effects for each variable for those treated and untreated with oseltamivir [6–9]. These heterogenous effects were also accounting for all other variables under study via the random forest approach and can be considered unbiased estimates of the absolute effect of the oseltamivir therapy conditioned on membership in a particular subgroup. Causal forests are extensions of the random forest, which split data repeatedly to create decision trees for classification or regression. The causal forest is built similarly, though instead of assessing the variable selection (splitting rules) based on prediction error, it maximizes the difference in treatment effects for the treatment/outcome pair. For the causal forest, a total of 50,000 trees were used to allow for accurate computation of 95% confidence intervals. Conditional average treatment effects were extracted for each categorical variable under consideration along with the 95% confidence interval and a data visualization was created for each outcome. R v4.04 (R Foundation for Statistical Computing, Vienna, Austria) was used for all analyses. The package *grf* was used for computation of causal forests and extraction of conditional average treatment effects [10].

## Results

A total of 691 hospitalized patients with lower respiratory tract infections randomized to either standard of care or standard of care plus oseltamivir with complete data for the variables under consideration were included in the study. In bivariable analyses (Table 1), two variables (Age category ≥ 75 years and Wednesday admission to the hospital) were significantly different across treatment status levels based on p-values, though q-values for all variables were 1, indicating statistically significant p-values were likely due to false discovery as opposed to true differences. Figure 1 depicts the conditional average treatment effect estimates for each variable under study for those treated with oseltamivir versus those who were not treated. For the primary outcome of clinical failure, patients who were documented influenza positive were the only subgroup identified with significant oseltamivir impact. The results identified a 26% lower occurrence of clinical failure for patients with influenza treated with oseltamivir versus those not treated with oseltamivir (95% CI 3.2–48.0%).

**Table 1:** Bivariable analysis comparing each of the selected subgroups to study arm

Demographics	Treated with oseltamivir	Not treated with oseltamivir	p-value	q-value
	n = 343	n = 348		
	n (%)	n (%)		
Age group 18–49 years	57 (16.6)	67 (19.3)	0.422	1.000
Age group 18–49 years	136 (39.7)	116 (33.3)	0.100	1.000
Age group 65–74 years	84 (24.5)	70 (20.1)	0.197	1.000
Age group ≥ 75 years	66 (19.2)	95 (27.3)	0.016	1.000
Male gender	202 (58.9)	196 (56.3)	0.305	1.000
Nursing home resident	17 (5.0)	19 (5.5)	0.899	1.000
Signs and symptoms				
Cough	330 (96.2)	325 (93.4)	0.135	1.000
Fever	145 (42.3)	146 (42.0)	0.994	1.000
Elevated white blood cell count	227 (66.2)	231 (66.4)	1.000	1.000
Past medical and social history				
Obese (BMI ≥ 30 kg/m^2^)	134 (39.1)	136 (39.1)	1.000	1.000
Active cancer	46 (13.4)	56 (16.1)	0.376	1.000
Congestive heart failure	88 (25.7)	102 (29.3)	0.322	1.000
Cerebrovascular accident	43 (12.5)	35 (10.1)	0.363	1.000
Renal disease	63 (18.4)	62 (17.8)	0.929	1.000
Liver disease	23 (6.7)	27 (7.8)	0.698	1.000
Chronic renal failure	51 (14.9)	48 (13.8)	0.768	1.000
Neurologic disease	83 (24.2)	78 (22.4)	0.642	1.000
Diabetes mellitus	120 (35.0)	131 (37.6)	0.517	1.000
History of community acquired pneumonia	62 (18.1)	57 (16.4)	0.624	1.000
Suspicion of aspiration	14 (4.1)	6 (1.7)	0.105	1.000
Cirrhosis	4 (1.2)	5 (1.4)	1.000	1.000
Alcoholism	8 (2.3)	9 (2.6)	1.000	1.000
History of COPD	200 (58.3)	210 (60.3)	0.640	1.000
Hospitalized ≥ 2 days in the prior 90 days	99 (28.9)	114 (32.8)	1.000	1.000
IV antibiotics in the past 90 days	88 (25.7)	89 (25.6)	0.311	1.000
Home infusion therapy	11 (3.2)	6 (1.7)	0.807	1.000
Home wound care	13 (3.8)	11 (3.2)	0.562	1.000
HIV disease	10 (2.9)	6 (1.7)	0.431	1.000
Statin use	133 (38.8)	145 (41.7)	0.486	1.000
Gastroesophogeal reflux diseases	40 (11.7)	53 (15.2)	0.207	1.000
Pulmonary edema due to congestive heart failure	3 (0.9)	10 (2.9)	0.098	1.000
Acute myocardial infarction on admission	8 (2.3)	6 (1.7)	0.766	1.000
Acute worsening of long-term arrhythmia on admission	8 (2.3)	5 (1.4)	0.558	1.000
Serious arrhythmia on admission	6 (1.7)	15 (4.3)	0.082	1.000
Antibiotic use in the prior 30 days	112 (32.7)	106 (30.5)	0.590	1.000
Home oxygen therapy	76 (22.2)	85 (24.4)	0.538	1.000
Frail	127 (37.0)	136 (39.1)	0.633	1.000
Unable to bathe	35 (10.2)	42 (12.1)	0.511	1.000
Unable to dress self	24 (7.0)	23 (6.6)	0.959	1.000
Unable to walk	40 (11.7)	43 (12.4)	0.870	1.000
Unable to get in and out of a chair	18 (5.2)	24 (6.9)	0.455	1.000
Unable to eat	8 (2.3)	12 (3.4)	0.517	1.000
Unable to use a toilet	18 (5.2)	32 (9.2)	0.063	1.000
Difficulty bathing	49 (14.3)	54 (15.5)	0.728	1.000
Difficulty dressing self	44 (12.8)	44 (12.6)	1.000	1.000
Difficulty walking	75 (21.9)	74 (21.3)	0.921	1.000
Difficulty getting in and out of a chair	49 (14.3)	45 (12.9)	0.683	1.000
Difficulty eating	22 (6.4)	17 (4.9)	0.480	1.000
Difficulty using toilet	43 (12.5)	31 (8.9)	0.156	1.000
Current smoker	114 (33.2)	101 (29.0)	0.265	1.000
History of influenza vaccine (current season)	238 (69.4)	243 (69.8)	0.966	1.000
Type and severity of disease				
Community-acquired pneumonia	236 (68.8)	228 (65.5)	0.402	1.000
Acute exacerbation of COPD	91 (26.5)	102 (29.3)	0.466	1.000
Acute bronchitis	16 (4.7)	18 (5.2)	0.894	1.000
Influenza positive	24 (7.0)	33 (9.5)	0.294	1.000
Arterial blood gas obtained	157 (45.8)	165 (47.4)	0.722	1.000
Altered mental status on admission	17 (5.0)	15 (4.3)	0.824	1.000
Need for ventilatory support on admission	32 (9.3)	36 (10.3)	0.749	1.000
Need for blood pressure support on admission	15 (4.4)	20 (5.7)	0.516	1.000
Less than 3 days of respiratory symptoms prior to admission	78 (22.7)	95 (27.3)	0.195	1.000
Need for ICU Care on Admission	45 (13.1)	52 (14.9)	0.562	1.000
Pneumonia severity index risk class IV or V	154 (44.9)	169 (48.6)	0.374	1.000
Radiographic findings				
Multilobar infiltrates on chest radiograph	119 (34.7)	114 (32.8)	0.647	1.000
Pleural effusion	58 (16.9)	73 (21.0)	0.205	
Right upper lobe infiltrate on chest radiograph	32 (9.3)	33 (9.5)	1.000	1.000
Right middle lobe infiltrate on chest radiograph	40 (11.7)	43 (12.4)	0.870	1.000
Right lower lobe infiltrate on chest radiograph	108 (31.5)	104 (29.9)	0.708	1.000
Left lower lobe infiltrate on chest radiograph	95 (27.7)	97 (27.9)	1.000	1.000
Unspecified lobe infiltrate on chest radiograph	15 (4.4)	15 (4.3)	1.000	1.000
Interstitial infiltrate on chest radiograph	26 (7.6)	18 (5.2)	0.254	1.000
Hospitalization and therapeutics				
Study year 1	81 (23.6)	88 (25.3)	0.672	1.000
Study year 2	169 (49.3)	172 (49.4)	1.000	1.000
Study year 3	93 (27.1)	88 (25.3)	0.646	1.000
Monday admission to hospital	49 (14.3)	50 (14.4)	1.000	1.000
Tuesday admission to hospital	60 (17.5)	58 (16.7)	0.851	1.000
Wednesday admission to hospital	70 (20.4)	49 (14.1)	0.036	1.000
Thursday admission to hospital	46 (13.4)	44 (12.6)	0.852	1.000
Friday admission to hospital	47 (13.7)	59 (17.0)	0.280	1.000
Saturday admission to hospital	36 (10.5)	41 (11.8)	0.677	1.000
Sunday admission to hospital	35 (10.2)	47 (13.5)	0.221	1.000
≤ 8 h from admission to antimicrobial therapy	269 (78.4)	271 (77.9)	0.933	1.000
Macrolide empiric therapy	144 (42.0)	148 (42.5)	0.946	1.000
Antithrombotic therapy during hospitalization	245 (71.4)	247 (71.0)	0.962	1.000
Systemic steroids during hospitalization	204 (59.5)	196 (56.3)	0.446	1.000
IV steroids on admission	170 (49.6)	169 (48.6)	0.852	1.000

## Discussion

This study suggests that addition of oseltamivir to standard of care may decrease clinical failure in hospitalized patients with influenza-associated lower respiratory tract infection versus standard of care alone. These results are reasonably consistent with the reductions in clinical failure with oseltamivir treatment in the per-protocol analysis from our original randomized trial which identified a non-significant reduction from 24% in the standard of care arm to 14% in the standard of care plus oseltamivir arm (p = 0.414) [4].

Causal inference in machine learning is a relatively new addition to the methodologic arsenal [5]. The causal forest, a special version of the generalized random forest [7], is the most widely used method for computing conditional average treatment effects in the medical literature; being used for readmission risk [11], targeted intervention development [6], diabetes epidemiology [12], and identification of risk factors for thyroid disease [13]. As discussed previously, causal machine learning methods are distinct from traditional supervised machine learning since these models estimate the treatment effect as opposed to risk or prediction. Further, they are not bound by parametric assumptions common in traditional methods such as regression modeling and are less apt to overfit through application of regularization in the computation [8, 11]. These factors, along with the ability to assess conditional average treatment effects in small sample sizes allowed us to perform a more robust analysis of these randomized data.

Here, the presence of influenza was the primary driver of significant decreases in clinical failure with oseltamivir therapy. This suggests that our approach to estimate conditional average treatment effects through causal machine learning methods is likely accurate and potentially more useful for detection of subgroup treatment effects in small samples. Future randomized trials may benefit from using similar methodologies as an adjunctive measure for the more traditional frequentist statistical methodologies typically utilized and reported. Use of these novel methods may assist in detection of subgroups where interventions are beneficial or detrimental, allowing for a movement toward more personalized medicine.

This study has several limitations. First, given the small sample size in the study, the variability in our treatment effect estimates is wide, as indicated by many of the 95% confidence intervals for many variables. Further, we were not able to assess model performance through training and testing given the small sample size, resulting in potentially biased results. Second, the generalizability of treatment effect estimates from causal forest methodologies has yet to be widely documented. These machine learning models for computation of heterogenous treatment effects have only begun to be utilized in medicine [6, 13, 14] and we were unable to find any studies using these methods in the field of respiratory infections. This study also used a composite outcome, combining several clinical outcomes: failure to reach clinical improvement within 7 days, transfer to intensive care 24 h after admission, or rehospitalization or death within 30 days. Because of this, we are not able to dissect which individual outcome is impacted by oseltamivir therapy. Further, given this is a novel computational approach for re-analysis of a single randomized clinical trial, we are unable to provide further clinical guidance on use of oseltamivir in the hospitalized patient and suggest continued application of national, regional, and local guidance on anti-influenza therapy.

The strengths of this study include both the data used from the largest randomized trial of hospitalized patients with oseltamivir therapy and the consistency of results from the initial trial using traditional methodologies such as regression modeling.

Future studies may benefit from these methods as adjunctive analytics in randomized trials and potentially for observational designs where appropriate adjustments can be made with collected data. By continuing to perform both methods, we can begin to identify the best analytic approaches to identify more targeted treatments to improve patient outcomes.

In conclusion, this secondary analysis of a randomized clinical trial suggests that oseltamivir may have clinical utility in hospitalized patients with influenza-associated lower respiratory tract infections. These results are supportive of current recommendations to initiate antiviral treatment in hospitalized patients with confirmed or suspected influenza as soon as possible after admission [3].

## Data Availability

The datasets generated and/or analyzed during the current study are not publicly available as they contain protected health information but may be available in de-identified format from the corresponding author on reasonable request.
